# Clinical characteristics, outcomes and prognostic factors in KRAS mutant lung cancers: experience from a tertiary care cancer center in India

**DOI:** 10.3332/ecancer.2024.1674

**Published:** 2024-02-22

**Authors:** Vanita Noronha, Laboni Sarkar, Vijay Patil, Nandini Menon, Minit Shah, Akash Pawar, Oindrila Roy Chowdhury, Omshree Shetty, Anuradha Chougule, Pratik Chandrani, Rajiv Kaushal, Trupti Pai, Amit Janu, Nivedita Chakrabarty, Kumar Prabhash

**Affiliations:** Tata Memorial Hospital, Mumbai 400012, India; † The authors contributed equally to the work.

**Keywords:** KRAS mutations, non-small cell lung cancer, real-world outcomes

## Abstract

**Objectives:**

Kirsten rat sarcoma viral oncogene homologue (*KRAS*) mutations in lung cancers, long considered untargetable, have had a recent rise in interest due to promising data of agents targeting *KRAS* p.G12C. As Indian data are scarce, we sought to identify baseline clinical characteristics, prognostic factors and outcomes of lung cancer patients with *KRAS* mutations at our hospital.

**Methods:**

Patients with KRAS mutant lung cancers treated at our institute from 2016 to 2022 were analysed.

**Results:**

133 patients with KRAS mutant lung cancers were identified. Median age was 57 (interquartile range 28–78) years, and 58 (43.6%) were smokers. 17 (12.7%) had brain metastases. The commonest variant was p.G12C, seen in 53 (39.8%) patients. Six (4.5%) had programmed death ligand 1 (PDL-1) expression >50% by Ventana SP263 PDL-1 assay, and 13 (9.7%) had epidermal growth factor mutation. Of 92 patients with available treatment details, the majority received intravenous chemotherapy, nine (9.8%) received tyrosine kinase inhibitors and four (4.4%) received immunotherapy (pembrolizumab). Median progression-free survival (PFS) with first-line therapy was 6 (95% confidence interval (CI) 2.8–9.2) months and median overall survival (OS) was 12 (CI 9.2–14.8) months. The incidence of brain metastases was higher in patients with G12C mutations (*p* = 0.025). Brain metastases (HR: 3.57, *p* < 0.001), Eastern Cooperative Oncology Group performance status (PS) ≥ 2 (HR: 2.13, *p* = 0.002) and G12C mutation (HR: 1.84, *p *= 0.011) were associated with inferior PFS, while brain metastases (HR: 4.6, *p* < 0.001), PS ≥ 2 (HR: 2.33, *p* = 0.001) and G12C mutation (HR: 1.93, *p* = 0.01) were associated with inferior OS.

**Conclusion:**

This is the largest dataset of KRAS mutant lung cancers from India. Brain metastases were higher in patients with G12C mutations and associated with poorer PFS and OS. G12C mutation and PS ≥ 2 were also associated with inferior PFS and OS. Experience with targeted therapy for KRAS mutations remains an area of future exploration due to the unavailability of these agents in India.

## Background

KRAS mutations were first identified in lung cancers more than three decades ago [1–3]. There has been a recent resurgence in interest in this subset of patients with lung cancer due to the development of agents targeting KRAS G12C, with promising phase 2 data for sotorasib [4, 5] leading to accelerated approval from the United States Food and Drug Administration in patients with locally advanced and metastatic KRAS mutant non-small cell lung cancer (NSCLC) who have progressed on a first-line agent. Most patients with KRAS mutations have a history of smoking and have had historically poorer prognoses relative to those with epidermal growth factor (EGFR) mutations. There is evidence for a lack of survival benefit from adjuvant chemotherapy in resected KRAS mutant lung cancers, and resistance to erlotinib or gefitinib [6–9].

In view of the paucity of data for Indian patients, we have sought to delineate baseline clinical characteristics, treatment courses, prognosis and outcomes of lung cancer patients with KRAS mutations at our hospital, a tertiary care cancer center in India. This article is being presented in accordance with the STROBE reporting checklist.

## Methods

This retrospective study involved patients with KRAS mutant lung cancers, identified from the database of patients with lung cancer from our institutional molecular tumour board clinic, wherein patients for whom next-generation sequencing (NGS) has been performed on baseline biopsy samples are discussed in a multidisciplinary meeting for therapeutic decision-making approaches at Tata Memorial Hospital. Clinical information was collected from individual patient electronic medical records including demographic data, baseline characteristics including smoking status, histopathology, radiological findings and clinical outcomes including toxicity assessment, progression, therapy at progression and survival. Patients for whom treatment records or follow-up data were unavailable and those with tumour histology other than non-small cell lung carcinoma and with EGFR and anaplastic lymphoma kinase (ALK) mutations were excluded from the survival analysis. The response was evaluated according to RECIST v 1.1. Toxicity was graded according to CTCAE, v5.0. Progression was defined as radiological progression, change of therapy or death from any cause. Computed tomography scans were done every 2–4 months or depending on patient’s symptoms. Progression-free survival (PFS) was calculated from the date of starting first-line therapy to disease progression or death. Overall survival (OS) was calculated from the date of registration to death from any cause.

### Molecular testing

Patients included had undergone NGS on formalin-fixed paraffin embedded samples using the institutional standard targeted gene panel SOPHiA Solid Tumor plus Solution, which identifies 139 RNA fusions, single nucleotide variants, gene amplification events in 24 genes, indels from 42 genes and microsatellite instability (MSI) status (six unique loci) following library preparation of the extracted DNA and RNA with paired end sequencing done on the Illumina MiSeq platform and data analysis using SOPHiA DDM software. Of patients who had undergone PDL-1 testing, this was performed by the Ventana SP263 PD-L1 assay.

### Statistical analysis

The statistical analysis was done using SPSS v 25. For descriptive statistics, continuous variables were reported as median with inter-quartile range (IQR) and categorical variables as frequencies and percentages. Pearson’s chi-square test was used to assess the association between two categorical variables. Two sample *z* tests were used to assess the difference between the proportions with the significance level set at 0.05. PFS and OS were evaluated by the Kaplan–Meier method. If the patient was lost to follow-up, the date of the last entry on their electronic medical records was taken as their last follow-up date. The Kaplan–Meier curve was plotted for the PFS and the OS over time in months [10, 11]. Log-rank test was used to compare the PFS and OS between different groups. The effect of covariates on survival was estimated using Cox proportional hazards model [12, 13].

## Results

**Figure 1.**
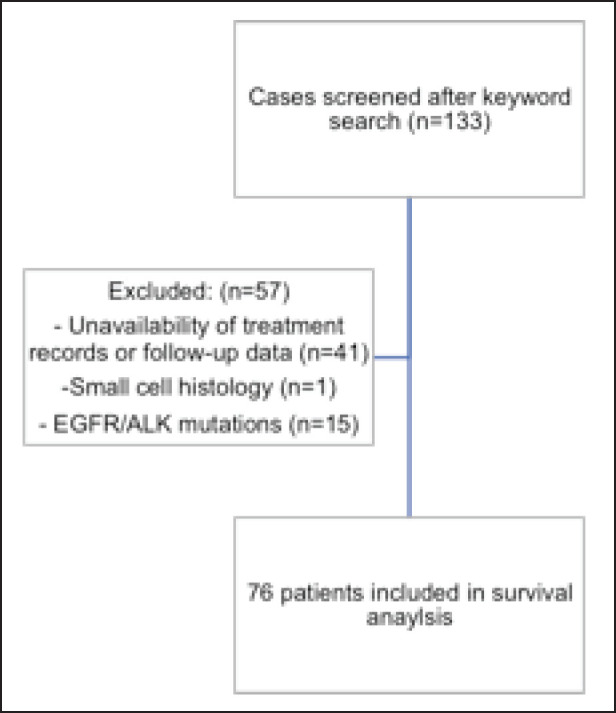
Identification of cases. A consort diagram detailing the patients screened after keyword search, with the number of patients excluded from the survival analysis and reasons for exclusion.

From September 2016 to July 2022, a total of 133 patients with KRAS mutant cancers were identified (Figure 1).

Demographic characteristics are detailed in Table 1. Median age was 57 (IQR 28–78) years. 87 (65.4%) patients were male. 58 (43.6%) had a history of smoking. Smokers had a mean of 33 (range 8–84) pack-years. Three patients had a history of intake of smokeless tobacco. 69 (51.9%) had an Eastern Cooperative Oncology Group (ECOG) performance status (PS) of 2 or above.

Clinical characteristics are detailed in Table 2. 117 (87.9%) patients had metastatic disease at baseline. The commonest site of metastases was lung, in 45 (33.8%) patients, followed by non-regional lymph nodes in 41 (30.8%) and pleural in 40 (30.1%). 17 (12.8%) patients had brain metastases. The mean tumour size was 57.5 mm (IQR 32.3–91.9).

The most frequent pathology was adenocarcinoma, seen in 111 (83.4%) patients, followed by squamous cell carcinoma in 18 (13.5%). The most common type of mutation was G12C, seen in 53 (39.8%). Other mutations were found in 54 (40.6%) patients – commonest among these was the TP53 mutation, found in 16 (12%) patients. 6 (4.5%) had PDL1 expression >50% by the Ventana SP263 assay, 13 (9.7%) had EGFR mutations and 2 (1.5%) had ALK rearrangements. Molecular and pathological characteristics are listed in Table 3. Serine/threonine kinase 11 (STK11)/liver kinase B1 (LKB1) and Kelch-like ECH-associated protein 1 (KEAP1) mutations were present in 29 (21.8%) and 16 (12.0%) patients, respectively.

The demographic, radiological, pathological and clinical characteristics of the tumours were also compared between G12C and non-G12C subtypes using the Pearson chi-square test. 25 of 53 (47.1%) patients in the G12C group and 33 of 80 (41.3%) patients in the non-G12C group had a history of smoking (*p* = 0.500). 11 (20.7%) of G12C mutant cases and 6 (7.5%) of non-G12C mutant cases had brain metastases (*p* = 0.025) (Table 4). Among the radiological and pathological characteristics, no significant difference was observed between the subtypes.

### Treatment details

Data on first-line treatment regimens were available for 92 patients, treated with non-curative intent. The commonest treatment modality was IV chemotherapy, received by 80 (86.9%) patients, most frequently used regimen being pemetrexed-carboplatin, used in 66 (71.7%) patients. This was combined with antiangiogenic targeted therapy (bevacizumab) in four patients, and immunotherapy (pembrolizumab) in four patients. Nine received first-line therapy with tyrosine kinase inhibitors. There were two patients with concomitant ALK rearrangements who received afatinib and ceritinib, and erlotinib and gefitinib were offered to four and three patients respectively on a compassionate basis. As sotorasib is unavailable in India, we were unable to offer this to our patients routinely. One patient received sotorasib for 6 months in the second-line setting after acquisition from abroad, with best response achieved being a partial response.

### Survival

**Figure 2.**
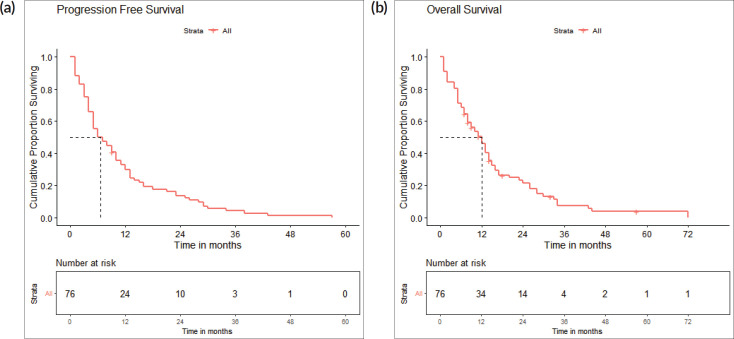
(a): Progression-free survival. A Kaplan-Meier curve depicting the PFS of all patients included in the survival analysis (*n* = 76). (b): Overall survival. A Kaplan-Meier curve depicting the OS of all patients included in the survival analysis (*n* = 76).

**Figure 3.**
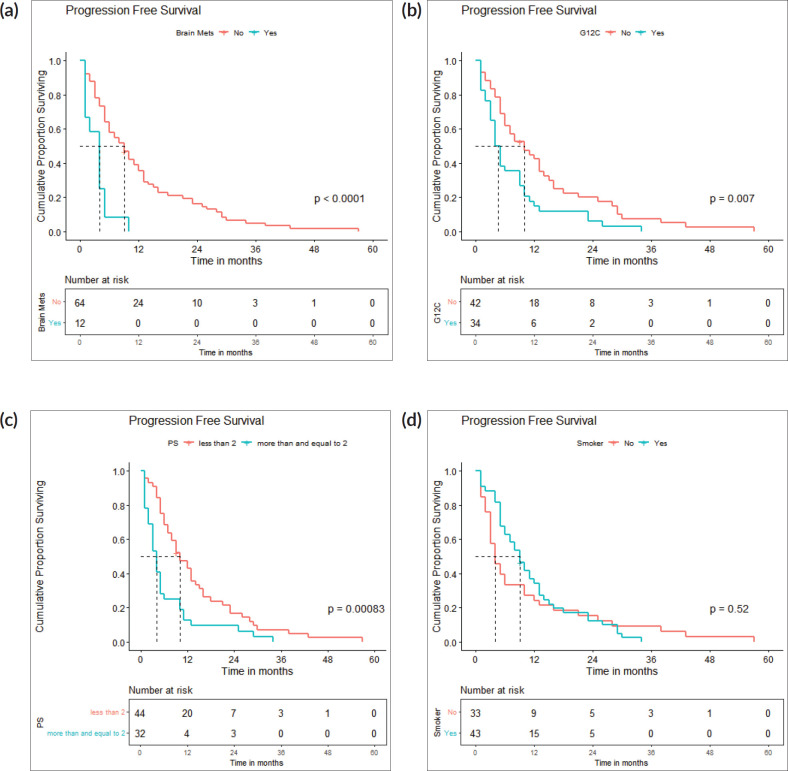
(a): PFS: brain metastases present versus absent. A Kaplan-Meier curve depicting the PFS of patients with (*n* = 12) and without (*n* = 64) brain metastases. (b): PFS: g12c versus other mutations. A Kaplan-Meier curve depicting the PFS of patients with (*n* = 34) and without (*n* = 42) G12C mutations. (c): PFS: PS <2 versus ≥2. A Kaplan-Meier curve depicting the PFS of patients included with ECOG PS <2 (*n* = 44) and ≥2 (*n* = 32). (d): PFS: smokers versus non-smokers. A Kaplan-Meier curve depicting the PFS of smokers (*n* = 43) and non-smokers (*n* = 33).

**Figure 4.**
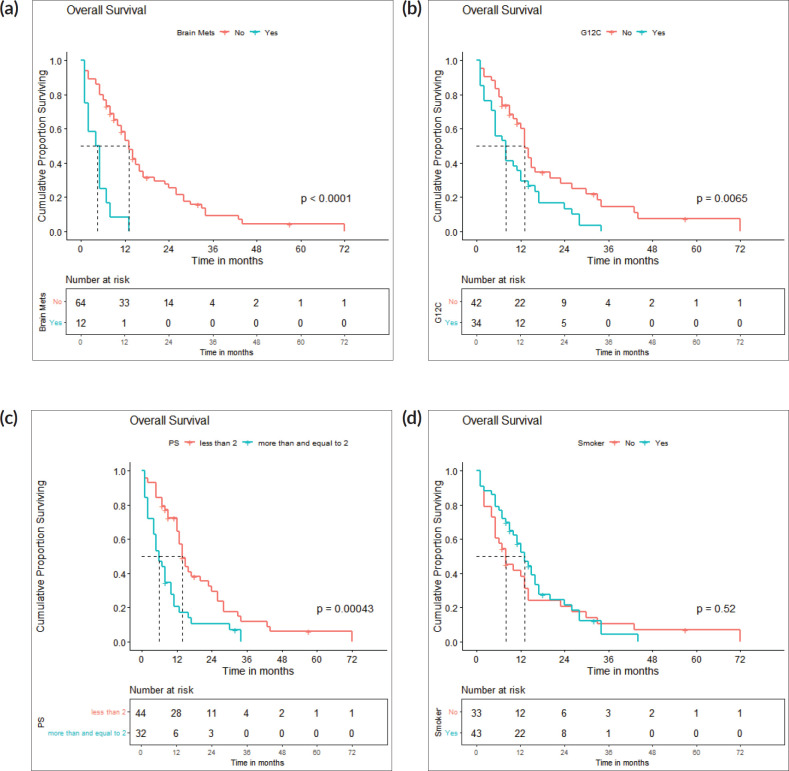
(a): OS: brain metastases present versus absent. A Kaplan-Meier curve depicting the PFS of patients with (*n* = 12) and without (*n* = 64) brain metastases. (b): OS: g12c versus other mutations. A Kaplan-Meier curve depicting the OS of patients with (*n* = 34) and without (*n* = 42) G12C mutations. (c): OS: PS <2 versus ≥2. A Kaplan-Meier curve depicting the PFS of patients included with ECOG PS <2 (*n* = 44) and ≥2 (*n* = 32). (d): OS: smokers versus non-smokers. A Kaplan-Meier curve depicting the OS of smokers (*n* = 43) and non-smokers (*n* = 33).

The survival analysis included 76 patients who received treatment for non-curative disease with available treatment and follow-up records, after excluding patients with EGFR and ALK mutations and non-NSCLC histology. With a median follow-up of 14.2 (IQR 2–71 months), median PFS with first-line chemotherapy was 6 (95% confidence intervals (CI), 2.8–9.2) months (Figure 2a). Median OS was 12 (CI 9.2–14.8) months (Figure 2b). There were 67 deaths by the date of data-cutoff time. Patients with brain metastases (*n* = 12) had a median PFS of 4 (CI 1–7) months and median OS of 4.5 (CI 2–11) months, while those without (*n* = 64) had a median PFS of 9 (CI 6–12) months and median OS of 13 (CI 11–16) months. Patients with G12C mutations (*n* = 34) had a median PFS of 4.5 (CI 4–9) months, while those with non-G12C mutations had a median PFS of 10 (CI 6–15) months (Figures 3a–d and 4a–d).

**Figure 5.**
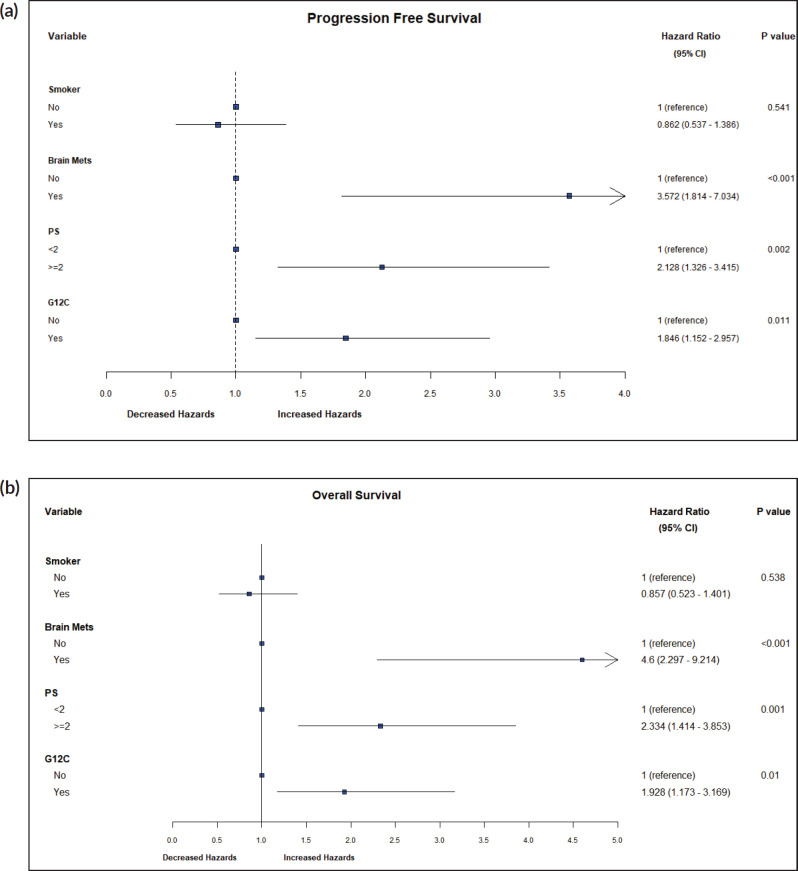
(a): Hazard ratios for PFS. A forest plot of hazard ratios for prognostic variables for PFS. (b): Hazard ratios for OS. A forest plot of hazard ratios for prognostic variables for OS.

By log-rank test, the presence of brain metastases (HR: 3.57, CI 1.81–7.03, *p* < 0.001), PS ≥ 2 (HR: 2.13, CI 1.33–3.4, *p* = 0.002) and G12C mutation (HR: 1.84, CI 1.15–2.96, *p* = 0.011) were associated with inferior PFS, while brain metastases (HR: 4.6, CI 2.29–9.21, *p* < 0.001), PS ≥ 2 (HR: 2.33, CI 1.41–3.85, *p* = 0.001) and G12C mutation (HR: 1.93, CI 1.17–3.17, *p* = 0.01) were associated with inferior OS (Figure 5a and b).

## Discussion

Our study sought to delineate baseline demographic, histopathological and radiological characteristics of patients with KRAS mutant lung cancers. The majority of the patients were male, and 43.6% were smokers. Existing literature suggests a correlation between KRAS mutations and race and smoking status, with patients being more commonly white and current or former smokers. The incidence of smoking in our population was not as high as in some studies, but still higher than the general incidence of smoking in patients with NSCLCs, which is around 15% [14]. Most patients had metastatic disease, with the most common sites of metastases being non-regional lymph nodes, lung, pleural and bone. Adenocarcinoma was the commonest histology, and the majority of tumours were poorly differentiated. The most common type of mutation was G12C, seen in 39.8% patients. This is consistent with data from other studies, which have reported G12C mutation incidence rates of 33%–45% [15–17].

We described radiological characteristics of KRAS mutant lung cancers in our hospital and compared these findings between G12C and non-G12C subtypes. We did not find a statistically significant difference between the groups based on mutation subtype. G12C KRAS mutant lung cancers have been reported to be likely to have a cavitary primary tumour with a higher frequency of lung metastases [18]. Relative to NSCLC with fusion rearrangements, a lower frequency of pleural metastases and lymphangitic carcinomatosis have been reported in G12C KRAS mutant lung cancers; brain and soft tissue metastases are, however, more common. The most common sites of metastases in our cohort were the lung, non-regional lymph nodes and pleura. Brain metastases were present in 12.7% of our patients overall, significantly higher in the G12C group (20.7% versus 7.5%). In a previous study, the most common sites of metastases in KRAS mutant lung cancers were bone, brain and lungs [18]. Yang *et al* [19] demonstrated that KRAS mutations were risk factors for brain metastasis in male patients with lung adenocarcinomas. The higher incidence of brain metastases in the G12C KRAS subgroup is clinically relevant and suggests these patients may benefit from closer monitoring for the development of neurological signs and/or symptoms and underlines the need for treatment modalities with reliable efficacy and penetration beyond the blood–brain barrier.

KRAS mutations have demonstrated a poorer OS compared to KRAS wild-type NSCLC [20]. In our study, the median PFS with first-line chemotherapy was 6 months, with a median OS of 12 months. This is consistent with previous reports [21–25].

Results on the impact of specific codon and point mutations on survival are variable. In a study of 677 patients with KRAS mutated advance stage NSCLC, those with mutations in KRAS codon 13 (*n* = 53) had poorer outcomes in comparison to codon 12 (*n* = 624), with a difference in survival of 2 months (*p* = 0.008), after adjusting for age, sex and smoking status [26]. Another analysis of 450 patients with KRAS mutated, metastatic adenocarcinoma lung found no OS difference between codon 12 and 13 mutations, although those with codon 13 mutations had a numerically lower 2-year survival [27]. Other studies demonstrated genotypic differences in survival, with G12C and p.G12V mutations being reported to have poorer survival relative to other subtypes [28]. One Asian study of 75 patients with advanced NSCLC reported improved PFS for patients with KRAS p.G12C mutations, particularly when treated with first-line pemetrexed-based chemotherapy [29]. In our population, G12C mutation was associated with inferior PFS and OS, consistent with most previous studies. Brain metastases and PS ≥ 2 were also predictive of poor survival, consistent with existing literature. There are conflicting data regarding the prognostic implication of KRAS mutations in earlier-stage lung cancers [30–32]. The small number of patients in our cohort receiving curative intent therapy precludes analysis.

Our study found no significant survival differences between smokers and non-smokers, although some studies have reported superior OS for never-smokers compared to current or former smokers [33]. This difference may be due to the lack of homogeneity among subgroups, with KRAS mutations being commoner in smokers, and EGFR and ALK mutations more common in non-smokers. When adjusting for the specific mutation, no survival difference between current/former/never smoking status has been demonstrated [34].

KRAS mutations may coexist with other master genes. The commonest co-occurring mutation in our cohort was TP53 mutation. In a Lung Cancer Mutation Consortium study, an additional carcinogenic driver was identified in a third of patients with KRAS mutations [27]. These mutations may have prognostic implications. Skoulidis *et al* [35] reported superior objective response rates for NSCLC with co-existent TP53 mutations in comparison with those with KRAS mutations alone (28.6% and 35.7%, respectively).

The majority of patients in our study received intravenous chemotherapy, with only a few being offered immunotherapy [36]. The low uptake of immunotherapy in our population is primarily due to financial challenges. The role of immunotherapy in the first-line treatment of patients with KRAS-mutant NSCLC is well established. Blocking the PD-1/PD-L1 pathway has been promising, with randomised phase II and III trials demonstrating improvement of OS in KRAS mutant NSCLC with checkpoint inhibitors compared to standard chemotherapy, as well as a recent meta-analysis reporting an OS improvement with anti-PD-(L)1 agents with or without chemotherapy in KRAS-mutant NSCLC in the first-line setting [37–40]. Authors have hypothesised the potential use of KRAS mutation status as a predictive biomarker in the selection of patients for immune checkpoint inhibitors as no significant OS benefit was observed for patients with KRAS wild type cancers. Due to the small number of patients receiving immunotherapy in our patient population due to financial constraints, a larger population and longer follow-up is needed to establish results.

Our study is limited by the small sample size, retrospective nature and unavailability of complete treatment records of a considerable number of our subjects. The need remains for appropriately powered studies with larger sample sizes to enhance our understanding of clinical, radiological and pathological characteristics of KRAS mutant lung cancers as well as therapeutic implications.

## Conclusion

This is the largest dataset of KRAS mutant lung cancers from India. Nearly half were smokers. 12.7% had brain metastases, significantly more in patients with G12C mutations and were associated with poorer PFS and OS. G12C mutation, the commonest subtype, was associated with inferior PFS, consistent with previous studies. Experience with targeted therapy for KRAS mutations remains an area of future exploration due to the unavailability of these agents in India.

## Conflicts of interest

There is no conflict of interest to disclose.

## Funding

This research did not receive any specific grant from funding agencies in the public, commercial, or not-for-profit sectors. The authors are accountable for all aspects of the work in ensuring that questions related to the accuracy or integrity of any part of the work are appropriately investigated and resolved.

## Author contributions

Vanita Noronha: conceptualisation, methodology, data curation, resources and supervision; writing – review and editing; Laboni Sarkar: conceptualisation, methodology and data curation; formal analysis; writing – original draft preparation; and writing – review and editing; Vijay Patil: data curation and resources; Nandini Menon: data curation and resources; Minit Shah: data curation and resources; Akash Pawar: methodology and formal analysis; Oindrila Roy Chowdhury: methodology and formal analysis; Omshree Shetty: data curation and resources; Anuradha Chougule: data curation and resources; Pratik Chandrani: data curation and resources; Rajiv Kaushal: data curation and resources; Trupti Pai: data curation and resources; Amit Janu: data curation and resources; Nivedita Chakrabarty: data curation and resources; and Kumar Prabhash: conceptualisation, methodology, data curation, resources and supervision; writing – review and editing.

## Figures and Tables

**Table 1.** Demographic characteristics.

Demographic characteristics	Median	Range	Number (%), *n* = 133
Age	57	28–78	
Gender			
Male			87 (65.4%)
Female			46 (34.6%)
Smoking status			
Smoker			58 (43.6%)
Non-smoker			75 (56.4%)
Performance status			
0–1			64 (48.1%)
≥2			69 (51.9%)

**Table 2.** Tumour characteristics.

Tumour characteristics	Number (%), *n* = 133
Stage	
II	1 (0.7%)
III	15 (11.3%)
IV	117 (87.9%)
Sites of metastases	
Non-regional lymph nodes	41 (30.8%)
Pleural	40 (30.1%)
Liver	9 (6.7%)
Lung	45 (33.8%)
Bone	30 (22.5%)
Adrenal	12 (9.0%)
Brain	17 (12.8%)

**Table 3.** Pathological and molecular characteristics.

Pathological and molecular characteristics	Number (%), (*n* = 133)
Histology	
Adenocarcinoma	111 (83.4%)
Squamous cell carcinoma	18 (13.5%)
Lymphoepithelioma	1 (0.7%)
Papillary carcinoma	1 (0.7%)
Pleomorphic carcinoma	1 (0.7%)
Small cell carcinoma	1 (0.7%)
Differentiation	
Well-differentiated	5 (3.7%)
Moderately differentiated	17 (12.7%)
Poorly differentiated	96 (72.2%)
Undifferentiated	15 (11.2%)
KRAS mutation type	
G12C	53 (39.8%)
G12V	27 (20.3%)
G12D	12 (9%)
G12A	11 (8.3%)
Q61H	3 (2.2%)
Others	27 (20.3%)
PDL1	
<1%	98 (73.7%)
1%–49%	29 (21.8%)
≥50%	6 (4.5%)

**Table 4.** Correlation between clinicopathological characteristics and type of KRAS mutation (*n* = 133).

	G12C mutation (*n* = 53)	Non-G12C mutation (*n* = 80)	*p*-value
Gender			
Male	35	52	0.901
Female	18	28	
Smoking status			0.500
Smokers	25	33	
Non-smokers	28	47	
Performance status			0.111
0–1	30	34	
≥2	23	46	
Brain metastases			0.025
Present	11	6	
Absent	42	74	
Liver metastases			0.771
Present	4	5	
Absent	49	75	
Bone metastases			0.985
Present	12	18	
Absent	41	62	
PDL1 status			0.983
<1%	39	59	
≥1%	14	21	
